# HERG1 functions as an oncogene in pancreatic cancer and is downregulated by miR-96

**DOI:** 10.18632/oncotarget.2200

**Published:** 2014-07-15

**Authors:** Jin Feng, Junbo Yu, Xiaolin Pan, Zengliang Li, Zheng Chen, Wenjie Zhang, Bin Wang, Li Yang, Hao Xu, Guoxin Zhang, Zekuan Xu

**Affiliations:** ^1^ Department of General Surgery, the First Affiliated Hospital of Nanjing Medical University, Nanjing 210029, China; ^2^ Department of Gastroenterology, the First Affiliated Hospital of Nanjing Medical University, Nanjing 210029, China; ^3^ Department of Gastroenterology, the First Affiliated Hospital of Nanchang University, Nanchang 330006, China

**Keywords:** pancreatic cancer, HERG1, miR-96, anticancer therapy

## Abstract

Pancreatic cancer is an aggressive malignancy with an extremely poor prognosis. The human ether-a-go-go-related potassium channel (HERG1) is a human rapid delayed rectifier, which is involved in many crucial cellular events. In this article, we find that HERG1 expression is dramatically increased both in pancreatic cancer tissues and cell lines, and that increased HERG1 expression is significantly related to the development of pancreatic cancer. HERG1 silencing in pancreatic cancer-derived cell lines PANC-1 and CFPAC-1 strongly inhibits their malignant capacity *in vitro* as well as tumorigenicity and metastasis in nude mice. In addition, HERG1 is identified as a direct target of miR-96, which is downregulated in pancreatic cancer tissues and cell lines. Ectopic expression of miR-96 represses the HERG1 expression in pancreatic cancer and significantly inhibits malignant behavior of pancreatic cancer cells *in vitro* and *in vivo*.

Collectively, our findings suggest that miR-96 acts as a tumor suppressor in pancreatic cancer and may therefore serve as a useful therapeutic target for the development of new anticancer therapy.

## INTRODUCTION

Pancreatic cancer is one of the most aggressive malignancies with an extremely low 5-year survival rate [1-2]. It is the sixth leading cause of death from malignant disease in China, and the fourth leading cause of cancer-related death in the United States [3-5]. The majority of patients by the time of diagnosis develop an aggressive form of disease which limits the potential for therapeutic intervention [6]. At this stage, several genetic and epigenetic changes have taken place, such as overexpression of oncogene KRAS and silencing of tumor-suppressor genes INK4A, TP53, and DPC4/Smad4 [7-9]. In addition, despite extensive research efforts, the prognosis for pancreatic cancer is the worst among all cancers due to minimal improvements in its prevention and treatment [3]. Therefore, the quest for new associated factors and novel therapeutic targets in pancreatic cancer remains an imperative clinical issue.

In 1996, Wonderlin and Strobel found that plasma membrane potassium (K^+^) channels are required for cell proliferation and have essential roles in every living cell [10]. More so, in recent years, an increasing number of studies found that ion channels, including K^+^, Na^+^, Cl^−^, and Ca^2+^ channels, are crucial for tumor development and progression [11-14]. The human ether-a-go-go-related potassium channel (HERG1), also known as KCNH2 or Kv11.1, is a human rapid delayed rectifier in the voltage-gated potassium channel family [15]. There is accumulating evidence that HERG1 protein is overexpressed in many types of human cancers [16-20], and is involved in many crucial cellular events such as proliferation, apoptosis, and invasion [18-21]. Nevertheless, studies examining the role of HERG1 in human pancreatic cancer are rare. Indeed, only recently it was suggested that HERG1 expression might affect the cell migration and proliferation in pancreatic ductal adenocarcinoma (PDAC) [22]. In addition, the mechanisms regulating HERG1 expression in tumor progression are still unclear.

MicroRNAs (miRNAs) are highly conserved endogenous small 20-25 nucleotide non-coding RNAs [23-24]. More than 50% of the known miRNAs have been shown to participate in human tumorigenesis and/or metastasis by directly targeting oncogenes or tumor suppressor genes [25-26]. For example, a miR-17-92 cluster was found to be located in a region that is commonly amplified in multiple human cancers and one of its targets is E2F, a transcription factor that is associated with DNA replication and apoptosis [27]. MiR-146a and miR-146b have been shown to have regulatory functions in the NF-kB pathway through their role in down-regulation of IL-1 receptor-associated kinase 1 and TNF receptor associated factor 6 protein levels [28]. More so, it has been suggested that some miRNAs might have contradictory roles depending upon the tumor type. Indeed, it has been shown that miR-96 (7q32.2) can function either as oncogene or tumor suppressor gene in different tumor types [29-33].

In the present study, we aimed at taking a comprehensive picture of the role of HERG1 in human pancreatic cancer through the analysis of its expression and function *in vitro* and *in vivo*. More so, we have examined the potenetial miR-96 role in the regulation of HERG1 function which may give further insights into our understanding of how miRNAs act in tumorigenesis and suggest novel therapeutic strategies in pancreatic cancer.

## RESULTS

### HERG1 expression in pancreatic cancer

**Figure 1:**
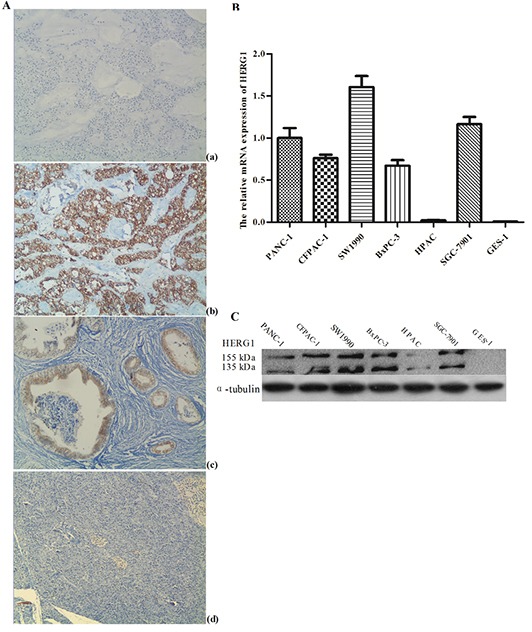
HERG1 was highly expressed both in pancreatic tumor tissues and cell lines **(A)** Immunohistochemical staining of HERG1 protein in pancreatic cancer, magnification, ×200: (a) negative control (PBS) (b) poorly differentiated pancreatic tumor tissues with positive HERG1 immunohistochemical reaction (c) well differentiated pancreatic tumor tissues with positive HERG1 immunohistochemical reaction (d) adjacent normal tissues negative for HERG1; **(B)** Semiquantitative RT-PCR analysis of HERG1 mRNA expression in pancreatic cancer cell lines: (a) agarose gel representing HERG1 mRNA positive (PANC-1, CFAC-1, SW1990, BxPC-3 and SGC-7901) and negative (HPAC and GES-1) mRNA expression (b) HERG1 mRNA expression in pancreatic cancer cell lines relative to the average HERG1 mRNA expression in normal pancreatic tissue adjacent to tumors; **(C)** Western blot analysis of HERG1 protein expression in pancreatic cancer cell lines: PANC-1, CFAC-1, SW1990, BxPC-3 and SGC-7901 with positive and HPAC and GES-1 with negative protein expression.

To test the HERG1 expression in pancreatic cancer and adjacent normal tissues, immunohistochemistry was performed in 78 pairs of pancreatic tissue samples. Positive immunohistochemical reaction was present mainly in the cytoplasm and membrane. HERG1 protein was highly expressed in pancreatic tumors, but weakly expressed in adjacent normal tissues (Fig. 1A). RT-PCR analysis (Fig. 1B) and western blot (Fig. 1C) revealed that both mRNA and protein HERG1 expression were markedly higher in pancreatic cancer cell lines PANC-1, SW1990, CFPAC-1, and BxPC-3 compared to the average HERG1 expression in normal tissues adjacent to the analyzed tumors. No HERG1 expression was detected in HPAC and GES-1 cell lines.

Further analysis of the clinicopathological characteristics in 78 pancreatic tumors showed that HERG1 staining was significantly higher in poorly-differentiated tumors compared to well-differentiated tumors. With respect to the TNM stage, HERG1 expression was much higher in stage II than in stage I tumors. In addition, a significant difference in the HERG1 expression was observed between tumors with lymph node involvement compared to those without lymph node involvement (Table 1).

**Table 1:** HERG1 protein expression and clinic- pathological features of pancreatic tumors

clinico-pathological features		HERG1 expression (positive/total number of analyzed samples)	*P*
**Sex**	male	44/49	0.621
	female	27/29	
**Age (y)**	≥65	36/41	0.295
	<65	35/37	
**Lymph node metastasis**	+	59/62	0.012
	−	12/16	
**TNM stage**	I	3/6	0.008
	II	68/72	
**Differentiation**	1. poor	41/41	1 vs. 3 0.000
	2. moderate	27/30	2 vs. 3 0.004
	3. well	3/7	1+2 vs. 3 0.001

*P*< 0.05 was considered as statistically significant.

### HERG1 silencing inhibits pancreatic cancer progression *in vitro*

**Figure 2:**
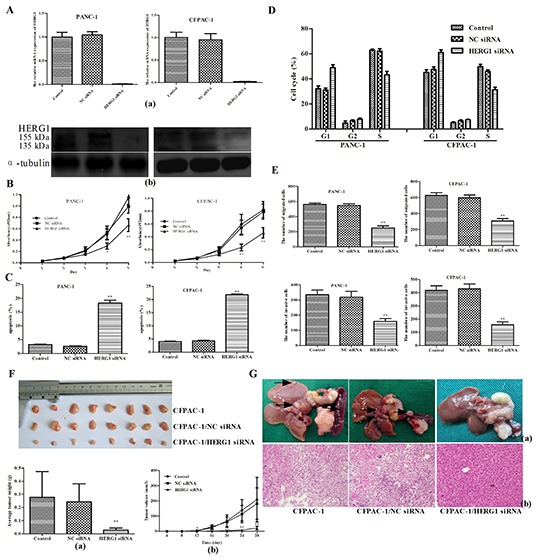
HERG1 siRNA silencing inhibits pancreatic cancer progression *in vitro* and *vivo* **(A)** HERG1 gene expression silencing reduced HERG1 mRNA (a) and protein (b) expression in PANC-1 and CFPAC-1 cells; **(B)** PANC-1 and CFPAC-1 growth curve after HERG1 siRNA, NC siRNA transfection or without treatment (MTT assay). The growth index was assessed at 1, 2, 3, 4, and 5 d; **(C)** Flow cytometery analysis of apoptosis in PANC-1 and CFPAC-1 cells after HERG1 siRNA, NC siRNA transfection or without treatement; **(D)** Flow cytometery analysis of cell cycle progression in PANC-1 and CFPAC-1 cells after HERG1 siRNA, NC siRNA transfection or without treatement; **(E)** Migration and invasion assay analysis of PANC-1 and CFPAC-1 cells after HERG1 siRNA, NC siRNA transfection or no treatement. **(F)** The growth curve (a) and weight of tumors (b) derived from the untreated, NC siRNA-transfected, and HERG1 siRNA-transfected CFPAC-1 cells injected into the left flank of 4-week-old female BALB/c nu/nu mice. **(G)** Metastatic nodules (a) and haematoxylin and eosin staining (H&E) (b) derived from the untreated, NC siRNA-transfected, and HERG1 siRNA-transfected CFPAC-1 cells injected into the pancreatic capsule of 4-week-old female BALB/c nu/nu mice. Magnification for H&E is ×40. All data are shown as mean ± SD. * *P* < 0.05; ** *P* < 0.01.

To study the HERG1 role in pancreatic cancer cell lines, siRNA silencing of HERG1 was applied and stable HERG1 siRNA transfected cell lines (PANC-1/HERG1 siRNA, CFPAC-1/HERG1 siRNA) were established (Fig. 2A). The HERG-1 silencing was confirmed by qRT-PCR and western blot at the mRNA and protein level, respectively.

To assess the proliferation of transfected cell lines, MTT assay was performed. The results of the MTT assay showed that proliferation of PANC-1/HERG1 siRNA cells was significantly slower than that of PANC-1 and PANC-1/NC siRNA transfected cells (Fig. 2B). The similar results were obtained for the CFPAC-1/HERG1 siRNA transfected cells.

Apoptosis analysis showed that HERG1 siRNA transfection increased the number of apoptotic cells. In comparison with the untreated cells, the cells transfected with HERG1 siRNA increased the percentage of cells entering apoptosis (~15.2% in PANC-1 and 17.8% in CFPAC-1; Fig. 2C; supplementary Fig. S1A).

Cell cycle analysis further confirmed this observation, indicating that HERG1 siRNA transfection induced cell cycle arrest in the G1 phase with a significant increase in the percentage of cells in this phase (~16.7% in PANC-1 and ~16.1% in CFPAC-1). More so, it induced a reduction of the S-phase cell population by ~18.3% (PANC-1) or ~19.7% (CFPAC-1) (Fig. 2D; supplementary Fig. S1B).

Furthermore, we have examined whether HERG1 could regulate the cell migration and invasion capability of pancreatic cancer cells. It is known that cell proliferation may interfere with the results of migration and invasion assay. Nevertheless, although HERG1 siRNA silencing caused a dramatic inhibition of cell proliferation, no major difference in cell proliferation was observed within the first 24 h. As shown in Fig. 2E, supplementary Fig. S1C the migration and invasion were significantly reduced in HERG1 siRNA transfected cells compared with the negative control in 24 h (*P* < 0.01).

### Stable depletion of HERG1 suppresses tumorigenicity and metastasis of pancreatic cancer cells *in vivo*

Furthermore, to examine whether HERG1 is linked to the pancreatic cancer progression *in vivo*, CFPAC-1, CFPAC-1/NC siRNA and CFPAC-1/HERG1 siRNA cells were injected into the left flank or pancreatic capsule of 4-week-old female BALB/c nu/nu mice. The experiment was performed only with CFPAC-1 cells since PANC-1 cells could not grow tumors in nude mice.

As shown in Fig. 2F, tumor growth was significantly slower in the CFPAC-1/HERG1 siRNA-transfected mice than in the empty vector-transfected and untreated controls. When tumors were removed from the mice (28 days after injection) the average weights of tumors derived from CFPAC-1/HERG1 siRNA clones were significantly lower (0.029 ± 0.015 g) than those derived from mice injected with CFPAC-1/NC siRNA (0.243 ± 0.138 g) and CFPAC-1 cells (0.278 ± 0.196 g).

In the pancreatic capsule injection experiment metastatic nodules in HERG1 siRNA transfected cells were significantly fewer than that in the negative control. (Fig. 2G)

### HERG1 is a direct target of miR-96

**Figure 3:**
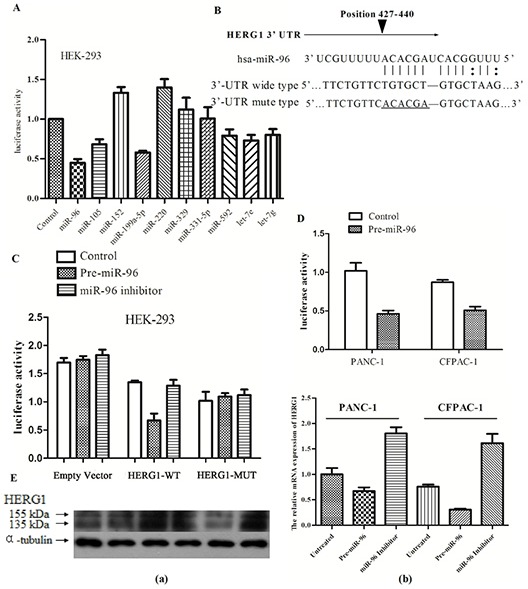
HERG1 is a direct target of miR-96 **(A)** Influence of miRNAs predicted to target HERG1 on luciferase reporter activity upstream of the HERG1 3'-UTR region. **(B)** The nucleotide sequence of miR-96 binding sites within the HERG1 3'-UTR region and a schematic diagram of the reporter constructs showing the entire HERG1 3'-UTR sequence (wild type, HERG1-WT and mutant type, HERG1-MUT); mutated nucleotides in miR-96 binding site are underlined. **(C)** HERG1-WT and the HERG1-MUT reporter luciferase activity in HEK-293 cells treated with pre-miR-96 or miR-96 inhibitor (50 nM), or without treatement. **(D)** HERG1-WT luciferase reporter activity in PANC-1 and CFPAC-1 after treatment with 50 nM of pre-miR-96, or without treatement. **(E)** HERG1 mRNA and protein expression in PANC-1 and CFPAC-1 cells treated with pre-miR-96 or miR-96 inhibitor, or without treatement. (a) Western blot (b) RT-PCR. All data are shown as mean ± SD.

In order to identify potential miRNAs targeting the HERG1 3'-UTR region we applied three different bioinformatic algorithms. As a result of this analysis, ten miRNAs were identified (the detailed information on these miRNAs is supplied in the supplementary Table S1. Next, we wanted to determine which miRNA is the most potent regulator of HERG1 gene levels. In brief, we co-transfected every pre-miRNA precursor with the reporter plasmid carrying the wild-type 3'-UTR region of HERG1 into HEK-293 cells, and as shown in Fig. 3A, we found the miR-96 is the most potent regulator of HERG1 gene levels *in vitro*. Based on these findings, we hypothesized that HERG1 might be a direct target of miR-96. In order to verify this hypothesis, in addition to constructing the reporter plasmid carrying the wild-type HERG1 3'-UTR region, we also constructed a reporter plasmid carrying the mutant-type HERG1 3'-UTR region (Fig. 3B). The HEK-293 cells were then transfected with either pre-miR-96 precursor or miR-96 inhibitor. As a result, pre-miR-96-transfected cells showed a marked reduction of wild-type reporter luciferase activity. Conversely, inhibition of luciferase activity by pre-miR-96 precursor was almost abolished in the mutant-type, suggesting that the predicted binding region was fully responsible for miR-96 function (Fig. 3C).

Furthermore, we repeated the experiments in human pancreatic cancer cell lines (PANC-1, CFPAC-1) with similar results (Fig. 3D). This led us to examine whether miR-96 ectopic expression could alter HERG1 mRNA and protein levels in human pancreatic cancer cell lines. Therefore we transfected pre-miR-96 precursor or miR-96 inhibitor into PANC-1 and CFPAC-1 cells and examined the influence of this treatment on HERG1 mRNA and protein levels. As expected, overexpression of miR-96 reduced the HERG1 expression, and conversely, in cells treated with miR-96 inhibitor, HERG1 expression was similar or higher than in the untreated controls (Fig. 3E). Collectively, these data suggest that HERG1 is a direct target of miR-96 in pancreatic cancer cell lines. Thus, downregulation of miR-96 in pancreatic cancer abolishes its role in HERG1 suppresion, which in turn might accelerate tumorigenesis.

### Aberrant miR-96 expression in pancreatic cancer

**Figure 4:**
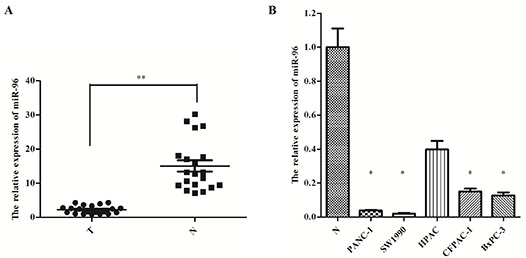
MiR-96 expression in pancreatic cancer tissues and cell lines **(A)** Real-time PCR-analysis of miR-96 expression in pancreatic tumors and matched adjacent non-tumor tissues and **(B)** pancreatic cancer cell lines. * *P* < 0.05; ** *P* < 0.01.

To determine whether aberrant miR-96 expression is associated with pancreatic cancer, we have examined miR-96 expression in 20 pairs of pancreatic cancer tissues and their matched adjacent non-tumor tissues using the TaqMan real-time PCR. The miR-96 expression was significantly lower in pancreatic cancer tissues compared to the adjacent non-tumor tissue (Fig. 4A).

Furthermore, we examined the miR-96 expression in five pancreatic cancer cell lines (PANC-1, SW1990, HPAC, CFPAC-1, and BxPC-3) and found that miR-96 expression was lower in tumor cell lines compared to the average miR-96 expression in adjacent non-tumor tissue, as well (Fig. 4B). These results suggest that miR-96 could have a tumor suppressing role in pancreatic cancer development and progression.

### MiR-96 decreases the proliferation, migration and invasion of pancreatic cancer cells *in vitro*

**Figure 5:**
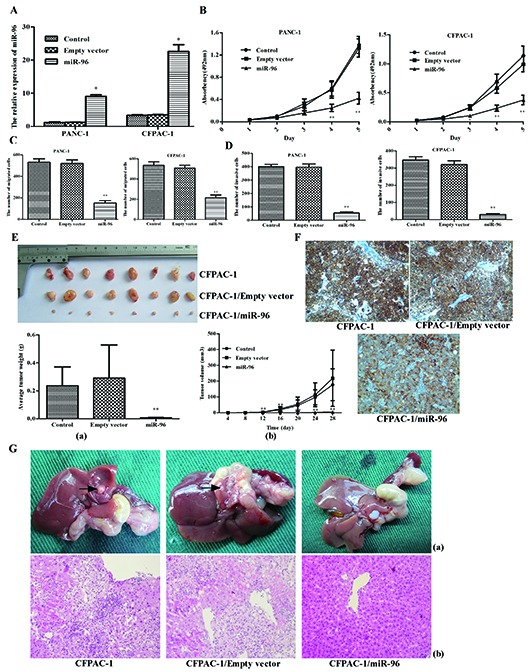
Overexpression of miR-96 inhibits pancreatic cancer cell growth, migration, invasion *in vitro*, suppresses tumorigenicity, metastasis and contrasts with HERG1 expression *in vivo* **(A)** plasmid induced overexpression of miR-96 in PANC-1 and CFPAC-1 cells; **(B)** PANC-1 and CFPAC-1 growth curve after transfection with pre-miR-96 plasmid or empty vector or without treatment. The growth index was assessed at 1, 2, 3, 4, and 5 d; **(C)** migration and **(D)** invasion assay analysis of PANC-1 and CFPAC-1 cells after transfection with pre-miR-96 plasmid or empty vector or without treatement. **(E)** The growth curve (a) and weight of tumors (b) derived from untreated, empty vector and miR-96 plasmid-transfected CFPAC-1 cells were injected into the left flank of 4-week-old female BALB/c nu/nu mice. **(F)** Immunohistochemical analysis of HERG1, in tumors derived from miR-96 overexpression, untreated and empty vector nude mice groups. **(G)** Metastatic nodules (a) and H&E staining (b) derived from untreated, empty vector and miR-96 plasmid-transfected CFPAC-1 cells injected into the pancreatic capsule of 4-week-old female BALB/c nu/nu mice. Magnification, ×40. All data are shown as mean ± SD. * *P* < 0.05; ** *P* < 0.01.

Previous results have verified that HERG1 is a direct target of miR-96 and therefore we wanted to further assess the biological role of miR-96 in pancreatic cancer cell lines. Our results on miR-96 expression in in PANC-1 and CFPAC-1 cells transfected with pre-miR-96 plasmid show that miR-96 expression was higher than in the untransfected controls (Fig. 5A). In addition, pre-miR-96 plasmid transfection of PANC-1 and CFPAC-1 cells significantly repressed cell proliferation, as shown by the MTT assay (Fig. 5B).

To further assess whether miR-96 is associated with progression of pancreatic cancer, we analyzed the effect of miR-96 expression on cell migration and invasion in PANC-1 and CFPAC-1 cell lines. The results of this experiment showed that pre-miR-96 transfection was associated with significant reduction in cell migratory and invasive capacity (P <0.01; Fig. 5C,; supplementary Fig. S2). Based on these findings, it seems that miR-96 overexpression diminishes pancreatic cancer cell migratory and invasive potential *in vitro*.

### MiR-96 suppresses tumorigenicity, metastasis and HERG1 expression *in vivo*

Finally, we wanted to examine whether miR-96 up-regulation reduced pancreatic cancer cell tumorigenicity and metastasis *in vivo* using the nude mice model. For this purpose, untreated, empty vector-transfected and miR-96 plasmid-transfected CFPAC-1 cells were injected into the left flank of 4-week-old female BALB/c nu/nu mice. For the duration of the treatment, tumor volumes were calculated every 4 days. Twenty-eight days after the initial treatement, the mice were sacrificed and tumors dissected and photographed. The results of the *in vivo* experiment show that tumor growth was significantly slower in miR-96 plasmid-transfected mice compared to the empty vector-transfected and untreated controls. The average weights of tumors derived from CFPAC-1/miR-96 (0.007 ± 0.002 g) clones were significantly smaller than those derived from the CFPAC-1/empty vector (0.291 ± 0.237 g) and untransfected CFPAC-1 cells (0.236 ± 0.135 g) (Fig. 5E).

Next, we analyzed HERG1 expression in nude mice tumor tissues by immunhistochemistry. The miR-96 plasmid-transfected group demonstrated the lowest level of HERG1 expression among the three groups (Fig. 5F). This result indicates that miR-96 can suppress the HERG1 expression *in vivo* and further demonstrates that HERG1 is a direct target of miR-96.

To examine whether miR-96 is linked to cancer metastasis *in vivo*, a nude mice pancreatic capsule injection experiment was used. Macroscopically, all mice developed tumors in their pancreas, but no metastatic nodules were observed in the mice injected with CFPAC-1/miR-96 clones (Fig. 5G).

## DISCUSSION

Pancreatic cancer is one of the most aggressive malignant diseases, and its development involves successive accumulation of mutations in a number of genes. Several major genetic changes have been described in pancreatic cancer including overexpression of oncogene KRAS and silencing of tumor-suppressor genes INK4A, TP53, and DPC4/Smad4 [7-9]. Recent advances in pancreatic cancer research aimed at improving patients’ survival have, so far, been disappointing, suggesting that new treatment strategies must be developed. The potassium channels have long been suggested to be related to the regulation of a variety of biological functions ranging from the control of cell excitability to the regulation of cell proliferation and migration [34-35]. Indeed, HERG1 K^+^ channel, a human rapid delayed rectifier in the voltage-gated potassium channel family, has been reported to be involved in the development and progression of many human cancers [16-20].

Our study is the first to show that HERG1 acts as a highly expressed oncogene in both human pancreatic cancer tumors and cell lines, and is involved in the progression of pancreatic cancer. It is also the first study to demonstrate that HERG1 gene is negatively regulated by miR-96 via a specific target site (nt427-440) within the 3'-UTR region of the HERG1 gene.

More so, clinicopathological analysis has revealed that HERG1 protein expression is significantly correlated with the TNM stage, tumor differentiation and lymph node metastasis. These findings are in agreement with the previous study in which HERG1 expression was in correlation with tumor grade in colorectal cancer [18].

In our study, HERG1 silencing inhibited proliferation, invasion and migration ability of pancreatic cancer cells, which is in line with the effects of HERG1 observed in other cancer types [18-21].

Furthermore, it has been demonstrated that HERG1 is involved in different phases of cell cycle progression [36-38]. Indeed, in our experiments, we found that pancreatic cancer cells transfected with HERG1-siRNA were arrested in G1 phase. Our results are similar to those of Shao and Wu who found that tumorigenesis was dramatically reduced in SGC7901 cells transfected with HERG1-siRNA [17].

In addition, in the present study, we have demonstrated that stable depletion of HERG1 suppresses tumorigenicity of CFPAC-1 in BALB/c nude mice. More so, metastatic nodules derived from the HERG1 siRNA transfected cells were significantly fewer than that in the negative controls.

It is becoming increasingly evident that miRNAs play important roles in the regulation of cell proliferation, apoptosis, migration and invasion thereby affecting normal cell growth and development and leading to a variety of disorders including malignancies [39-41]. In our study, we have identified several miRNAs abnormally expressed in pancreatic cancer, such as miR-21, miR-301a and miR-155. Indeed, it has been shown in recent studies that these miRNAs are involved in regulating development of pancreatic cancer via specific target genes (PDCD4, NF-kappaB, TP53INP1) [42-44].

For these reasons, we investigated the possibility that miRNAs are involved in HERG1 upregulation. A computer search for miRNA targets in the HERG1 3'-UTR sequence revealed several potential miRNAs complementary to this region, among which miR-96 was the strongest candidate. Indeed, our *in vitro* luciferase assay study showed that miR-96 was the most potent regulator of the HERG1 gene.

Expression of miR-96 in cancer is controversial because it has been found to be either downregulated [45-47] or upregulated in tumors [48-50]. In the present study, we have shown that miR-96 is a new, evolutionarily conserved regulator of HERG1, which is significantly downregulated in pancreatic cancer. These results suggest that miR-96 functions as a tumor suppressor with a potential role in pancreatic cancer tumorigenesis. These results are in line with the results of a previous study on pancreatic ductal adenocarcinomas [33].

Next, we wanted to examine whether HERG1 is a direct target of miR-96. In our study, miR-96 overexpression or inhibition significantly downregulated or upregulated HERG1 protein and mRNA levels, respectively. In addition, miR-96 overexpression was associated with the suppression of luciferase-HERG1-3'UTR activity, indicating that HERG1 is, indeed, a direct target of miR-96.

Furthermore, in this study, we found that overexpression of miR-96 significantly reduced proliferation, migration and invasion of pancreatic cancer cells *in vitro*. In the *in vivo* experiments, tumor growth curves and the average weight of the tumors revealed a significant decrease in tumor growth rates following the treatment with the miR-96. More so, a significant decrease in metastatic nodules was also observed. These results indicate that miR-96 is an important tumor suppressor miR in pancreatic cancer and that upregulation of this miRNA in low-expressing pancreatic cancer cells decreases their malignant potential. These results are consistent with the results of a previous study in which miR-96 was shown to be a tumor suppressor and a potent regulator of the KRAS gene [51]. Taken together, our results show that miR-96 may be considered as a novel therapeutic target in pancreatic cancer treatment.

In conclusion, our study shows that HERG1 is an important and often upregulated oncogene in pancreatic cancer development and progression. Furthermore, we have shown that its expression is directly regulated by miR-96. More so, overexpression of miR-96 can inhibit the malignant capacity of pancreatic cancer cells via the HERG1 regulation. Based on these results we can speculate that restoration of miR-96 expression in pancreatic cancer might provide a novel therapeutic strategy for pancreatic cancer.

## MATERIALS AND METHODS

### Cell lines and human tissue samples

The human pancreatic cancer cell lines (PANC-1, SW1990, CFPAC-1, HPAC, BxPC-3), gastric cancer cell lines (GES-1, SGC-7901), and human embryonic kidney cell line (HEK-293) were provided by Laboratory of Gastroenterology, the First Affiliated Hospital of Nanjing Medical University. The cells were cultured in DMEM (Invitrogen, Carlsbad, CA, USA) containing 10% fetal bovine serum (Hyclone, Milan, Italy) and antibiotics (penicillin 100 units/ml and streptomycin 100 ug/ml) at 37°C in a humidified atmosphere with 5% CO2. For immunohistochemical detection, a total of 78 pairs of paraffin-embedded pancreatic cancer tissues and adjacent normal pancreatic tissues were obtained from the archive tissue bank of the Department of Pathology at Jiangsu Province Hospital, Nanjing, China. For miR-96 detection, 20 pairs of pancreatic cancer tissues and adjacent normal pancreatic tissues immediately snap-frozen in liquid nitrogen were obtained from patients undergoing surgery for pancreatic cancer in Jiangsu Province Hospital, and were stored at -80°C until RNA extraction. The study was approved by the Ethics Committee of the First Affiliated Hospital of Nanjing Medical University, and the number of the document was 2008(1101). Written informed consent was obtained from all subjects.

### Immunohistochemistry

Immunohistochemistry (IHC) was performed according to the previously described methods [52]. Briefly, paraffin-embedded sections were deparaffinized and rehydrated. Next, antigens were retrieved in citrate buffer, and the sections were blocked with normal rabbit serum followed by overnight incubation with anti-HERG1 antibody (1:50 dilution, Santa Cruz Biotechnology) at 4°C. The sections were further incubated with rabbit anti-goat IgG conjugated with horse radish peroxidase for 30 minutes at 37°C. After washing with PBS, the sections were treated with DAB at room temperature for four minutes and counterstained with haematoxylin. Negative controls were performed with PBS instead of the antibody. All sections were examined independently by two experienced pathologists.

### Reverse transcription and polymerase chain reaction amplification

Total RNA was extracted from cells and tissues with Trizol reagent according to the manufacturer's instructions (Invitrogen, Carlsbad, CA, USA). The RNA purity and quality were examined on 1% agarose gels. One to two μg of RNA were reverse transcribed (RT) with Superscript II (Invitrogen, Carlsbad, CA, USA), using 250 μM random hexamers in a 20 μl reaction mix. RNase inhibitor (1 U) (Roche) was used to avoid RNA degradation. The reaction was carried out following the manufacturer's instructions.

Total cDNA was used as a template for polymerase chain reaction amplification (PCR) with Herculase DNApol (Stratagene, Cedar Creek, TX). Primers and thermal cycling conditions are presented in the supplementary Table S2.

### Western blot analysis

For the Western blot (WB) analysis, cells were washed three times with ice-cold phosphate-buffered salt solution (PBS) and then lysed with RIPA buffer. Cell debris was removed by centrifugation at 12000 r/min for 20 min at 4°C, and protein concentration was determined using the Bradford method with BSA (Pierce, Rockford, IL, USA) as a standard. Protein samples (40 μg) were then separated on 10% sodium dodecyl sulfate-polyacrylamide gel electrophoresis (SDS-PAGE) and then transferred to Immobilon-P membranes (Millipore, Bedford, MA, USA). After 1 hour blocking with 5% non-fat milk in PBS-Tween-20, membranes were incubated with anti-HERG1 antibody (1:500 dilution) or monoclonal anti-α-tubulin (1:5000) antibody overnight at 4°C. After the Tris-Buffered Saline and Tween 20 (TBST) washing, membranes were incubated with the appropriate secondary antibody (1:5000) linked to a horse radish peroxidase for 1 hour at 37°C. Immunohistochemical reaction was detected using ECL Plus Detection kit (Pierce, Rockford, IL, USA).

### TaqMan real-time polymerase chain reaction detection of miR-96

The miR-96 level was quantified by quantitative real-time RT-PCR using Taqman assay kits (Applied Biosystems, Foster City, CA), with RNU6B small nuclear RNA as an internal reference. The analysis was performed using a 7900 Real-Time PCR System (Applied Biosystems) and the reaction mixture and parameters were set according to the manufacturer's instruction. All samples were run in triplicate.

### Plasmid vector construction and screening

In order to construct four HERG1 siRNA plasmids, four pairs of double-stranded siRNA oligonucleotides were inserted into the pcDNA™6.2-GW/EmGFPmiR vector (Invitrogen, Carlsbad, CA, USA). Oligonucleotide sequences of four different HERG1 small interfering RNAs (HERG1 siRNAs) and a negative control siRNA (NC siRNA) (Invitrogen, Carlsbad, CA, USA) that were used in this experiment are preseted in the supplementary Table S3. At 48 hours after transfection, the cells were collected for RNA extraction. Silencing efficiency of every siRNA was examined by real-time qPCR, and the results are shown in the supplementary Table S4.

To generate the pre-miR-96 plasmid construct, we amplified a 589 bp DNA fragment carrying a precursor miR-96 from PANC-1 genomic DNA using the pre-miR-96 forward and reverse PCR primers (supplementary Table S5), and inserted the fragment into the KpnI and XbaI site of the plasmid vector pcDNA3.1-GFP (Invitrogen, Carlsbad, CA, USA).

### Generation of stable transformants

For the generation of stable transformants, cells were seeded onto six-well plates and cultured in DMEM. Lipofectamine 2000 (Invitrogen, Carlsbad, CA, USA) was used to transfect HERG1 siRNA plasmid, pre-miR-96 plasmid, and respective negative control plasmids into cells at 60%-70% confluency. To select for positive colonies, cells were cultured for 48 hours after transfection in a selective medium containing 3 μg/ml blasticidin (Sigma-Aldrich, Saint Louis, MO, USA) and maintained for two weeks. After this period, 100 cells were plated on each 10-cm dish and after the following two weeks, colonies were selected and plated on 24-well plates. After a week, these cells were passaged onto six-well plates and finally stable transformants were identified by fluorescence microscopy, qRT-PCR, Western blot or real-time PCR.

### Construction of promoter reporter plasmids and luciferase assays

In order to identify potential miRNAs targeting the HERG1 3'-UTR region three bioinformatic algorithms (TargetScan, MicroCosm Targets and PicTar) were adopted. Among the potential miRNA candidates, ten miRNAs were chosen in order to find which one is the most potent regulator of HERG1 gene levels. In brief, the luciferase assay was performed and every pre-miRNA precursor with the reporter plasmid carrying the wild-type 3'-UTR region of HERG1 was cotransfected into HEK-293 cells.

As miR-96 was identified as the most potent regulator of HERG1 levels next it was examined whether HERG1 is a direct target of miR-96. In brief, the miR-96-binding site in the HERG1 3'-UTR region (wild or mutant-type) was cloned downstream of the firefly luciferase gene in a pGL3-promoter vector (Promega, Madison, WI). For the luciferase assay, cells were cultured in 12-well plates and contransfected with 50 nM of oligonucleotide, 200 ng of the luciferase reporter construct and 20 ng of the renilla luciferase reporter construct. After 48 h, luciferase activity was measured using the dual luciferase reporter assay system (Promega, Madison, WI).

### MTT assay

Cell proliferation was examined by MTT assay. Briefly, cells in the logarithmic growth phase were harvested and seeded on 96-well plates (Costar, Schiphol-Rijk, Netherlands). Cells were diluted to 1000/well in 200 μl DMEM. 20 μl of MTT (Sigma-Aldrich, Saint Louis, MO, USA) were added to each well 4 hours before culture termination. 200 μl of DMSO were added to each well at the end of the culturing period and the plates was agitated for 15 minutes. The absorbance at 490 nm was read by an automatic microwell plate reader (Bio-Rad 3350). Each assay was performed in triplicate.

### Cell migration and invasion assay

For the cell migration and invasion assays, 8 μm pore sized plain (for migration) or matrigel-coated (for invasion) transwell inserts (Corning Costar, Schiphol-Rijk, Netherlands) were placed in the wells of 24-well culture plates. The upper chamber (filter insert) was filled with 100 μl of cell suspension (1 × 10^5^ cells) in serum-free DMEM medium. The lower chamber contained 600 μl of DMEM with 10% fetal bovine serum. After 48 h incubation filters were fixed with methanol and stained with crystal violet. Cells remaining on the top side of the filter were removed by soft mechanical scraping and the number of cells migrating to the bottom of the filter was counted under a light microscope (in each chamber six fields were counted at 200x magnification).

### Cell cycle distribution

For the cell cycle distribution analysis, cells were harvested by centrifugation, washed with PBS and fixed overnight with ice-cold 75% ethanol at -20°C. The following day, fixed cells were centrifuged and washed with PBS. Each sample was then resuspended in propidium iodide stain buffer in PBS for 15 min in the dark at room temperature. After the staining, 2 × 10^4^ cells per sample were analyzed using a FACScan flow cytometer (Becton Dickinson; San José, CA, USA). Each assay was performed in triplicate.

### Apoptosis assay

For the apoptosis assay, cells were harvested by centrifugation and washed with PBS. Each sample was double stained with FITC-Annexin V and propidium iodide. FITC-Annexin V (Boehringer Mannheim, Mannheim, Germany) was added at a final concentration of 1 mg/ml, and then 10 mg/ml of propidium iodide were added. The mixture was incubated for 15 minutes in the dark at room temperature and the fluorescence was quantified on 1 × 10^4^ cells using a FACScan flow cytometer (Becton Dickinson; San José, CA, USA). Each assay was performed in triplicate.

### Animal models

For the *in vivo* study on animal models, cells in the logarithmic growth phase were harvested and resuspended in PBS. To analyze the tumorigenicity, 5 × 10^6^ cells in 0.2 ml suspension were injected subcutaneously into the left flank of 4-week-old female BALB/c nu/nu mice (purchased from the Nanjing Medical University; License No. SCXK (Su) 2008-0004)). Tumor volumes were measured with vernier caliper every 4 days after injection. The volume of tumors was calculated as volume = (length × width^2^) × 0.5. Tumors were dissected and photographed on day 28 after injection. For the metastasis assay, 2 × 10^6^ cells in 0.1 ml were injected into pancreatic capsule of 4-week-old female BALB/c nu/nu mice. Four weeks after injection, the mice were sacrified and their livers, stomachs, spleens and pancreas were removed and fixed in 4% formalin. Metastatic nodules larger than 1 mm were detected as a valuable focus. Experimental and control group consisted of at least eight mice each. Animal handling and experimental procedures were approved by the Nanjing Medical University animal experiments Ethics committee.

### Statistics

Data from at least three independent experiments were expressed as means ± standard deviation (SD). The differences between groups were analyzed using the Student's t-test when only two groups were present, or by one-way analysis of variance (ANOVA) when more than two groups were compared. The data were considered significant if *P* < 0.05 (indicated by “*”) and *P* < 0.01 (indicated by “**”).

## SUPPLEMENTARY FIGURES AND TABLES


