# Supplementary material for: HERG1 functions as an oncogene in pancreatic cancer and is downregulated by miR-96
Source: Oncotarget. 2014 Jul 15;5(14):5832–44. doi: 10.18632/oncotarget.2200 (PMC4170607; doi:10.18632/oncotarget.2200)
Supplement: Supplementary file 1 [file oncotarget-05-5832-s001.pdf]

SUPPLEMENTARY FIGURES AND TABLES

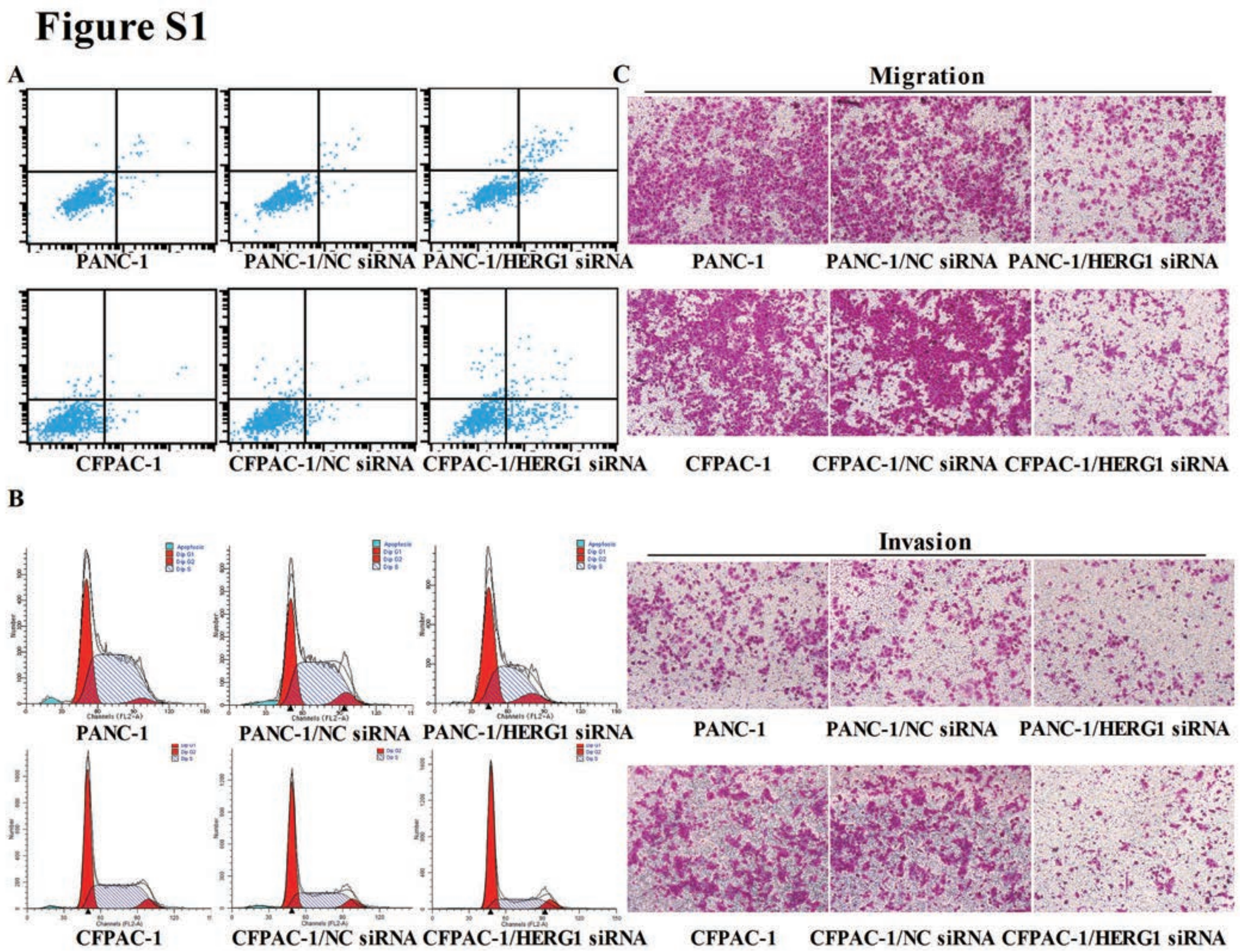

Supplementary Figure S1: HERG1 siRNA silencing inhibits pancreatic cancer progression *in vitro*. (A) Flow cytometery analysis of apoptosis in PANC-1 and CFPAC-1 cells after HERG1 siRNA, NC siRNA transfection or without treatement; **(B)** Flow cytometery analysis of cell cycle progression in PANC-1 and CFPAC-1 cells after HERG1 siRNA, NC siRNA transfection or without treatement; **(C)** Migration and invasion assay analysis of PANC-1 and CFPAC-1 cells after HERG1 siRNA, NC siRNA transfection or no treatement. Magnification, ×100.

Figure S2

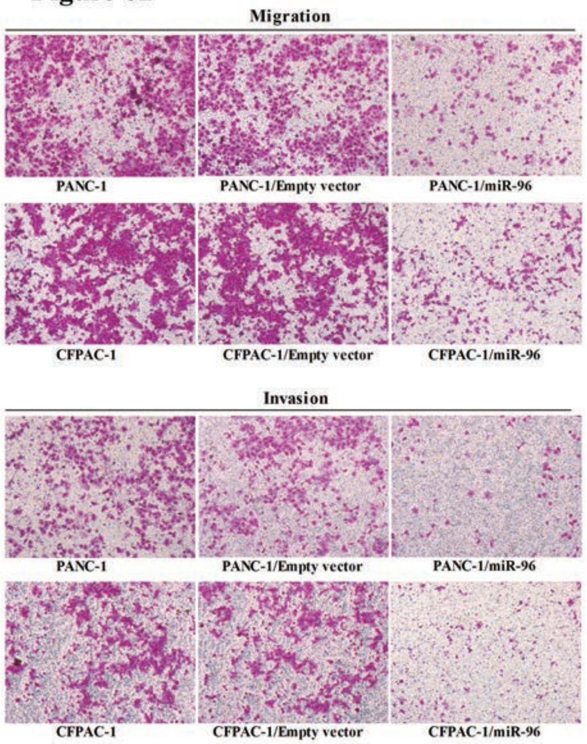

Supplementary Figure S2: Overexpression of miR-96 inhibits pancreatic cancer cell migration, invasion *in vitro*. Migration and invasion assay analysis of PANC-1 and CFPAC-1 cells after transfection with pre-miR-96 plasmid or empty vector or without treatement. Magnification, ×100.

Supplementary Table S1. The miRNAs potentialy targeting the 3'-UTR of HERG1

miRNAs<br>Position of HERG1 3'-UTR	miRNA (top) and predicted consequential pairing of target region (bottom)
hsa-miR-96<br>Position 427-440	ACACGAUCACGGUUU<br>TGTGCT—GTGCTAAG
hsa-miR-105<br>Position 60-68	UCGUAAACU<br>GGCATTTGG
hsa-miR-152<br>Position 419-425	AAGACAG<br>TTCTGTT
hsa-miR-199a-5p<br>Position 382-390	UUGUGACCC<br>AACACTGGG
hsa-miR-220<br>Position 193-199	GCCACAC<br>TGGTGTG
hsa-miR-329<br>Position 195-201	CCACACA<br>GGTGTGT
hsa-miR-331-5p<br>Position 90-95	AGGGAC<br>TCCCTG
hsa-miR-592<br>Position 116-123	AACUGUGU<br>TTGACACA
hsa-let-7e<br>Position 435-440	UUCGAU<br>AAGCTA
hsa-let-7g<br>Position 325-333	GGACAUGU<br>TCTGTACA

Supplementary Table S2. Primer pairs and thermal cycling conditions

**HERG1**	primers	5’-CGCACCATTAGCAAGATTCCC-3’<br>5’-AAGACAGCCGTGTAGATGACCAG-3’
	thermal cycling conditions	denaturating phase at 95°C for 5 min, 35 cycles of 1 min at 94°C, 1 min annealing phase at 58°C, and a 1 min extension phase at 72°C, with a final extension phase of 10 min at 72°C
**beta-actin**	primers	5’-TGCGTGACATTAAGGAGAAGC-3’<br>5’-GCCAGGGTACATGGTGGTG-3’
	thermal cycling conditions	denaturating phase at 95°C for 5 min, 35 cycles of 15 seconds at 95°C, 30 seconds annealing phase at 65°C, and 30 seconds extension phase at 58°C, with a final extension phase of 10 min at 72°C

Supplementary Table S3. Oligonucleotide sequences of four different HERG1 siRNAs and a negative control siRNA

Oligo name	oligo sequences 5'to 3'
HERG1 siRNA-1-F	TGCTGCGATGAGGTCCACCACAGCCAGTTTTGGCCACTGACTGACTG GCTGTGGGACCTCATCG
HERG1 siRNA -1-R	CCTGCGATGAGGTCCCACAGCCAGTCAGTCAGTGGCCAAAACTGGCT GTGGTGGACCTCATCGC
HERG1 siRNA -2-F	TGCTGATGTGTGGCTGCTCCATGTTGGTTTTGGCCACTGACTGACCAA CATGGCAGCCACACAT
HERG1 siRNA -2-R	CCTGATGTGTGGCTGCCATGTTGGTCAGTCAGTGGCCAAAACCAACA TGGAGCAGCCACACATC
HERG1 siRNA -3-F	TGCTGTGCTGTTGTAGGGTTTGCCTAGTTTTGGCCACTGACTGACTAG GCAAACTACAACAGCA
HERG1 siRNA -3-R	CCTGTGCTGTTGTAGTTTGCCTAGTCAGTCAGTGGCCAAAACTAGGC AAACCCTACAACAGCAC
HERG1 siRNA -4-F	TGCTGTTTGCAGTGCTGCAGCAGTGAGTTTTGGCCACTGACTGACT ACTGCTAGCACTGCAAA
HERG1 siRNA -4-R	CCTGTTTGCAGTGCTAGCAGTGAGTCAGTCAGTGGCCAAAACTCAC TGCTGCAGCACTGCAAAC
Negative-F	TgctgAAATGTACTGCGCGTGGAGACGTTTTGGCCACTGACTGACGTCT CCACGCAGTACATTT
Negative-R	CctgAAATGTACTGCGTGGAGACGTCAGTCAGTGGCCAAAACGTCTCC ACGCGCAGTACATTTc

Supplementary Table S4. Plasmid vector screening: silencing efficiency of four different HERG1 siRNAs and a negative control siRNA

HERG1	siRNA-1	siRNA-2	siRNA-3	siRNA-4	NC siRNA
ΔCt	13.4335	13.6975	13.3029	13.49777	11.18273
ΔΔCt	2.7465	3.0105	2.6159	2.810767	0
2-ΔΔCt	0.149012	0.124094	0.1631307	0.14252	1
Interferential efficiency	0.850988	0.875906	0.8368693	0.85748	0

Supplementary Table S5. The oligonucleotide sequences of pre-miR-96 forward and reverse PCR primers

Oligo name	oligo sequences 5'to 3'
pre-miR-96-F	TCTGGGTACCGCACTGGTAGAATTCACTG
pre-miR-96-R	CCTTTCTAGACCACGGCACCATTCAGGA
